# Description of the dermatoscopic features observed in sporotrichosis and American cutaneous leishmaniasis in a reference center in Rio de Janeiro, Brazil^☆^

**DOI:** 10.1016/j.abd.2022.09.015

**Published:** 2023-07-20

**Authors:** Alejandra Galeano España, Maria Inês Fernandes Pimentel, Janine Pontes de Miranda Lyra, Cláudia Maria Valete-Rosalino, Marcelo Rosandiski Lyra

**Affiliations:** a Laboratory of Clinical Research and Surveillance in Leishmaniasis, Instituto Nacional de Infectologia Evandro Chagas, Fundação Oswaldo Cruz, Rio de Janeiro, RJ, Brazil; b Department of Dermatology Center Izamar Millidiu Silva, Santo Cristo, Rio de Janeiro, RJ, Brazil; c Otolaryngology Department and Ophthalmology, Faculty of Medicine, Universidade Federal do Rio de Janeiro, Rio de Janeiro, RJ, Brazil

**Keywords:** Cutaneous, leishmaniasis, Dermoscopy, Infectious diseases, Sporotrichosis

## Abstract

**Background:**

The evaluation of American cutaneous leishmaniasis (CL) and sporotrichosis (SP) with dermoscopy may improve the diagnosis accuracy and clinical monitoring.

**Objectives:**

To describe the dermoscopic findings and patterns of skin lesions of patients with CL and SP followed up at the Laboratory of Clinical Research and Surveillance in Leishmaniasis (LaPClinVigiLeish), Evandro Chagas National Institute of Infectious Diseases (INI), Oswaldo Cruz Foundation, Rio de Janeiro, Brazil.

**Methods:**

The authors included patients with a diagnosis of CL or SP, who attended at INI/ Fiocruz, between 2019‒2021. All patients had 3 dermoscopic examinations (DermLite DL4): before treatment (T_0_), during treatment (T_1_), and after healing (T_2_). Up to three lesions per patient were evaluated.

**Results:**

The authors studied 47 patients with CL (74 lesions), and 19 patients with SP (24 lesions). The authors described dermoscopic structures such as rosettes, white lines, white dots, brown focal structureless areas, brown lines and dots, white perilesional circles, perilesional hyperchromic circles, microulcerations and the rainbow patterns. The authors created specific patterns; in CL: CL-T_0_ “central yellow scales with a white perilesional circle pattern”, CL-T_1_ “diffuse structureless white area pattern” and CL-T_2_ “white and brown focal structureless areas pattern”. In SP: SP-T_0_ the “pustule with erythema pattern”; SP-T_1_ the “focal structureless white areas with erythema pattern” and SP-T_2_ the “white linear pattern”.

**Study limitations:**

This study does not correlate dermoscopic findings with time of disease evolution at the first medical examination.

**Conclusions:**

The recognition of CL and SP dermoscopy patterns may be helpful tool for the differential diagnosis and monitoring of disease evolution.

## Introduction

American tegumentary leishmaniasis (ATL) is an infectious, non-contagious disease caused by different species of protozoa of the genus *Leishmania*, which affects the skin and/or mucous membranes in patients from the New World.^1^ Cutaneous leishmaniasis (CL) is the most frequent clinical manifestation. It usually appears as a papule that evolves into an ulcer or verrucous plaques.^2^ The diagnosis is confirmed by observation of the parasite.^3^ Sporotrichosis (SP) is a subacute or chronic infection caused by a dimorphic fungus: S*porothrix schenckii sensu lato*, a complex comprising several species. It is a polymorphic disease of the skin and subcutaneous tissue.^4^ It is an endemic disease in the state of Rio de Janeiro (RJ), Brazil, where the species *Sporothrix brasiliensis* predominates, and correlated with atypical clinical presentations of the disease.^5^ The gold standard for the diagnosis of SP is the isolation and identification of *Sporothrix sp*.^6^ In RJ, the main clinical differential diagnosis of CL is SP, particularly with ulcerated or verrucous fixed skin forms.^7^ Dermoscopy is a non-invasive technique to visualize the skin in vivo which adds information that could not be observed with the naked eye.^8^ Dermoscopy can be a useful tool in areas where different diseases with similar clinical presentations coexist, such as CL and SP in the state of RJ. Studies on CL dermoscopy are rare and concerning the old-world disease, this work brings a new and different perspective on ATL dermoscopy. Regarding SP, dermoscopy publications were limited to case reports. The present study aims to the recognition of dermoscopic patterns related to specific dermatological presentations of CL and SP (ulcerated, ulcerative-vegetative, verrucous, gummy, etc.), at different points in the follow-up of the patients.

## Methods

The authors evaluated a total of 74 lesions of CL (47 patients) and 24 lesions of SP (19 patients) at the Laboratory of Clinical Research and Surveillance in Leishmaniasis (LaPClinVigiLeish), Evandro Chagas National Institute of Infectious Diseases (INI), RJ, Brazil. Skin biopsies were performed with a 6 mm diameter punch at the edge of the lesions, and the diagnosis was confirmed in the material biopsied by observation of the parasite or the fungus, using at least one of the following methods: skin scraping or imprint, histopathological/immunohistochemical exam, culture, molecular exams (Polymerase Chain Reaction: PCR) or culture media (Sabouraud agar with chloramphenicol or Mycobiotic agar). The authors used the Dermlite DL4 dermoscope at 10-fold magnification and they chose polarized light for a better analysis of vascular structures. Clinical and dermoscopic photographs were taken at three points of follow-up: before the beginning of Treatment (T_0_), after 30 days of the beginning of Treatment (T_1_), and after healing (T_2_). The statistical analysis was performed with SPSS version 16.0 (IBM Corp, Armonk, NY, USA) comparing the dermoscopic features of both diseases with Pearson Chi-Square or Fisher's Exact Test. A descriptive study of the different global patterns for each disease was also performed.

## Results

The results of the dermoscopic study are shown in Table 1. At CL-T_0_, the most frequent dermoscopic findings were general erythema (82%); microulcerations (73%); focal structureless white areas (73%); hyperkeratosis (68%), white lines (58%), fiber sign (58%), central ulcer (49%), white dots (45%), focal structureless yellow areas (40%), white perilesional circle (37%), follicular plugs (32%) and rosettes (26%). At CL-T_1_, the most representative findings were: general erythema (92%); white lines (77%); focal structureless white areas (72%); white dots (68%); microulcerations (52%) and hyperkeratosis (50%). At CL-T_2_ there was a predominance of white findings related to the healing process as follows: white dots (79%); focal structureless white areas (79%); and white lines (61%), along with previously unpublished dermoscopic structures related to dyschromia of the CL healing process as brown focal structureless areas (62%) and black/brown dots (65%). At CL-T_2_ there was also the predominance of the rainbow pattern (44%). Different vascular patterns were observed in all phases of CL (Table 2): at CL-T_0_ the predominant pattern was polymorphic vessels (75%), followed by glomerular vessels (60%) and dotted vessels (56%). At CL-T_1_ glomerular vessels predominated (47%), followed by polymorphic vessels (45%) and dotted vessels (37%). At CL-T_2_ there was a predominance of polymorphic vessels (43%), followed by irregular linear vessels (36%), dotted vessels (25%), linear vessels with branches (28%), and glomerular vessels (28%).

**Table 1:** Comparison of dermoscopic features of Cutaneous Leishmaniasis Lesions (CL) and Sporotrichosis lesions (SP). Evandro Chagas National Institute of Infectious Diseases, Oswaldo Cruz Foundation, Rio de Janeiro, Brazil

Dermoscopy features	T_0_			T_1_			T_2_		
	CL n (%)	SP n (%)	p	CL n (%)	SP n (%)	p	CL n (%)	SP n (%)	p
General erythema	60 (82)	24 (100)	0.034*	57 (91.9)	15 (93,8)	1	44 (72.1)	14 (70)	0.767
Microulcerations	53 (72.6)	14 (60.9)	0.306	32 (51.6)	10 (62.5)	0.576	12 (19.7)	4 (20)	1
Focal structureless white areas	53 (72.6)	20(87)	0.178	45 (72.6)	15 (93.8)	0.099	48 (78.7)	17 (85)	0.749
Hyperkeratosis	50 (68.5)	11 (47.8)	0.86	31 (50)	11 (68.8)	0.18	13 (21.3)	4 (20)	1
White lines	42 (57.5)	11 (47.8)	0.475	48 (77.4)	15 (93.8)	0.175	37 (60.7)	14 (70)	0.596
Fiber sign	42 (57.5)	10 (43.5)	0.337	13 (21)	5 (31.2)	0.506	1 (1.6)	0	∼
Central ulcer	36 (49.3)	15 (65.2)	2.33	18 (29)	7 (43.8)	0.368	1 (1.6)	0	∼
White dots	33 (45.2)	11 (47.8)	1	42 (67.7)	14 (87.5)	0.211	48 78.7)	13 (68.4)	0.369
Focal structureless yellow areas	29 (39.7)	9 (39.1)	1	11 (17.7)	2 (12.5)	1	6 (9.8)	2 (10)	1
White perilesional circle	27 (37)	4 (17.4)	0.124	3 (4.8)	1 (6.2)	1	1 (1.6)	0	∼
Follicular plugs	23 (31.5)	6 (26.1)	0.796	16 (25.8)	6 (37.5)	0.365	7 (11.5)	2 (10)	1
Rosettes	19 (26)	4 (17.4)	0.426	16 (25.8)	7 (43.8)	0.219	19 (31.1)	7 (35)	0.786
Structureless focal brown areas	18 (24.7)	8 (34.8)	0.421	25 (40.3)	7 (43.8)	1	38 (62.3)	14 (70)	0.6
Bleeding	17 (23.3)	6 (26.1)	1	6(9.7)	1(6.2)	1	0	0	∼
Crust	15 (20.5)	4 (17.4)	1	18 (29)	7 (43.8)	0.368	3 (4.9)	1 (5)	1
Rainbow pattern	13 (17.8)	3 (13)	0.754	17 (27.4)	7 (43.8)	0.234	27 (44.3)	12 (60)	0.303
Pustule	12 (16.4)	9 (39.1)	0.04*	3 (4.8)	0	∼	0	0	∼
White starbust pattern	13 (17.8)	5 (21.7)	0.761	4 (6.5)	1 (6.2)	1	3 (4.9)	1 (5)	1
Yellow tears	11 (15.1)	0	∼	5 (8.1)	1 (6.2)	1	3 (4.9)	1 (5)	1
Black/brown dots	10 (13.7)	3 (13)	1	12 (19.4)	4 (25)	0.729	28 (45.9)	13 (65)	0.198
Perilesional hyperchromic circle	6 (8.2)	0	∼	11 (17.7)	2 (12.5)	1	10 (16.4)	3 (15)	1
Salmon-colored ovoid structures	4 (5.5)	1 (4.3)	1	9 (14.5)	1 (6.2)	0.678	13 (21.3)	4 (20)	1
Perilesional pigment	3 (4.1)	2 (8.7)	0.59	11 (17.7)	3 (18.8)	1	9 (14.8)	2 (10)	0.723
Inverted network	0	0	∼	2 (3.2)	0	∼	9 (14.8)	3 (15)	1
Strawberry pattern	5 (6.8)	2 (8.7)	0.672	4 (6.5)	0	0.576	2 (3.3)	0	∼
Brown lines	0	0	∼	0	0	∼	12 (19.7)	3 (15)	0.751
Comedon	0	0	∼	0	0	∼	0	8 (40)	∼

T_0_, Before the beginning of treatment; T_1_, Thirty days after the beginning of treatment; T_2_, After healing; CL, Cutaneous Leishmaniasis; SP, Sporotrichosis; p, p-value.

* means p with statistical difference.

**Table 2:** Comparison of vascular dermoscopic features of Cutaneous Leishmaniasis Lesions (CL) and Sporotrichosis lesions (SP). Evandro Chagas National Institute of Infectious Diseases, Oswaldo Cruz Foundation, Rio de Janeiro, Brazil

Vascular dermoscopy features	T_0_			T_1_			T_2_		
	CL n (%)	SP n (%)	p	CL n (%)	SP n (%)	p	CL n (%)	SP n (%)	p
Polymorphic vessel pattern	55 (75.3)	19 (82.6)	0.577	28 (45.2)	8 (50)	0.784	26 (42.6)	10 (50)	0.611
Dotted vessels	41 (56.2)	13 (56.5)	1	23 (37.1)	7 (43.8)	0.626	15 (24.6)	5 (25)	1
Glomerular vessels	44 (60.3)	13 (56.5)	0.81	29 (46.8)	8 (50)	1	17 (27.9)	7 (35)	0.544
Linear vessels	19 (26)	6 (26.1)	1	13 (21)	5 (31.2)	0.506	16 (26.2)	5 (25)	1
Linear vessels with branches	8 (11)	5 (21.7)	2.91	8 (12.9)	2 (12.5)	1	17 (27.9)	6 (30)	1
Corkscrew vessels	5 (6.8)	3 (13)	0.393	3 (4.8)	0	∼	0	0	∼
Comma vessels	2 (2.7)	1 (4.3)	0.565	8 (12.9)	2 (12.5)	1	1 (1.6)	0	∼
Irregular linear	25 (34.2)	15 (65.2)	0.14	7 (11.3)	2 (12.5)	1	22 (36.1)	8 (40)	0.793
Hairpin vessels	23 (32.5)	12 (52.2)	0.086	11 (17.7)	3 (18.8)	1	1 (1.16)	0	∼

T_0_, Before the beginning of treatment; T_1_, Thirty days after the beginning of treatment; T_2_, After healing; CL, Cutaneous leishmaniasis; SP, Sporotrichosis; p, p-value.

The main dermoscopic findings at SP-T_0_ were general erythema (100%), focal structureless white areas (87%), microulcerations (61%), central ulcer (65%), hyperkeratosis (48%), fiber sign (44%), pustule (39%), focal structureless yellow areas (39%) and white perilesional circle (17%). At SP-T_1_ the following dermoscopic findings decreased: erythema (63%), fiber sign (31%), and focal structureless yellow areas (13%). On the other hand, an increase of white dots (88%), hyperkeratosis (69%), microulcerations (63%), brown focal structureless areas (44%), rosettes (44%) and follicular plugs (38%) was observed. SP-T_2_ had a predominance of focal structureless white areas (85%), erythema (70%) white dots (68%), white lines (65%), black-brown dots (65%) and comedon (40%). The most frequent vascular patterns at SP-T_0_ were (Table 2): polymorphic vessels pattern (83%), irregular linear vessels (65%), glomerular vessels (57%), dotted vessels (57%) and hairpin-like vessels (52%). At SP-T_1_ was a predominance of polymorphic vessels (50%) glomerular vessels (50%), linear vessels (31%), and linear vessels with branches (13%). At SP-T_2_ polymorphic vessels pattern was the most common vascular finding (50%), followed by linear vessels with branches (30%) and linear vessels (25%).

**Figure 1:**
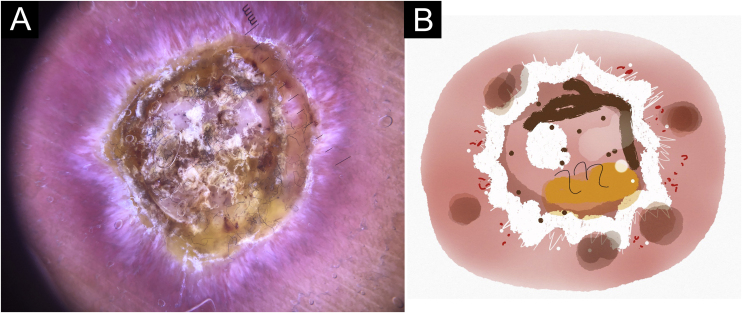
Cutaneous leishmaniasis ulcer crustose lesion of patient (n = 29) on the arm, at the initial medical appointment. (A) Dermoscopy. (B) Illustration of the dermoscopic pattern: “central yellow scales with white perilesional circle”. The “central yellow scales with white perilesional circle pattern” is composed of general erythema, central ulcer with hyperkeratosis, microulcerations, focal structureless white areas, white lines, white dots, polymorphic vessels (mostly glomerular vessels, dotted vessels), surrounding by white perilesional circle

**Figure 2:**
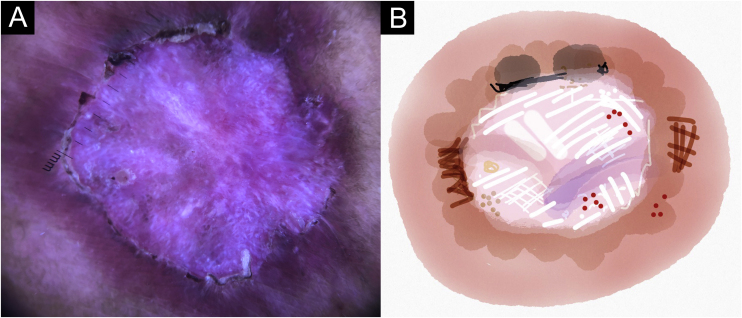
Cutaneous leishmaniasis epithelialized lesion of patient (n = 29), on the arm, thirty days after the beginning of the treatment. (A) Dermoscopy. (B) Illustration of the dermoscopic pattern: “diffuse structureless white area pattern”. The “diffuse structureless white area pattern” is composed of general erythema, focal structureless white area, white lines, white dots, microulcerations, polymorphic vessels and brown focal structureless areas

**Figure 3:**
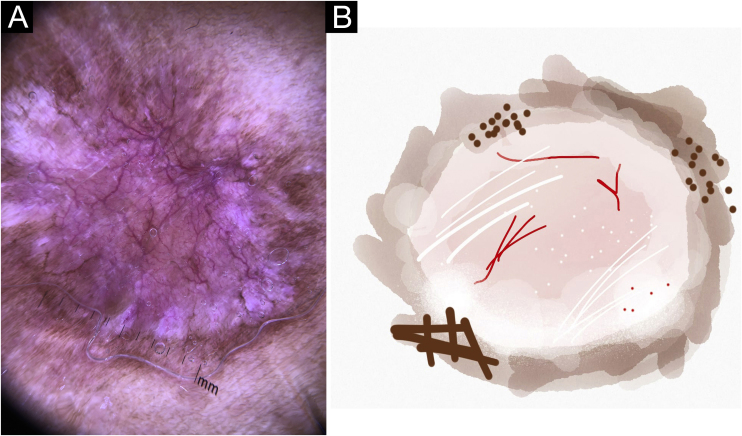
Cutaneous leishmaniasis dyschromic- atrophic cicatricial lesion of patient (n = 29) in the arm at cure. (A) Dermoscopy. (B) Illustration of the dermoscopic pattern “atrophic-hyperchromic”. The “white and brown focal structureless areas pattern” is composed of general erythema, focal structureless white area, white lines, white dots, polymorphic vessels (mostly linear irregular vessels), brown focal structureless areas, brown lines and black/brown dots

**Figure 4:**
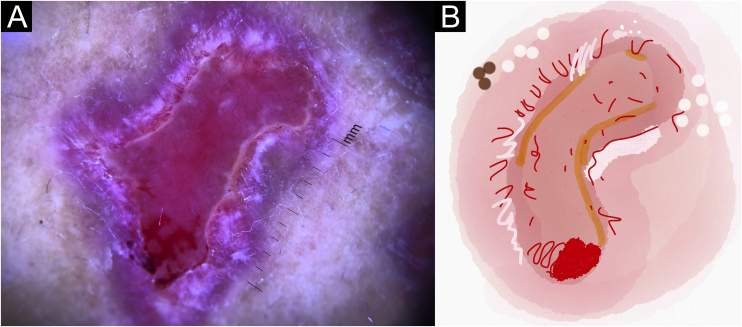
Sporotrichosis ulcerated plaque lesion of patient (n = 69) on the arm, at the initial medical appointment. (A) Dermoscopy. (B) Illustration of the dermoscopic pattern “pustule with erythema pattern”. The “pustule with erythema pattern” is composed of pustule, general erythema, central ulcer, microulcerations, focal structureless white areas, white lines, white dots, focal structureless yellow areas and polymorphic vessels (mostly linear irregular vessels and hairpin like vessels)

**Figure 5:**
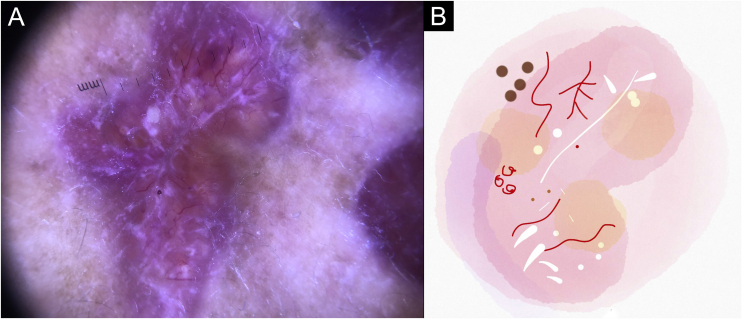
Sporotrichosis lesion of patient (n = 69) on the arm, thirty days after the beginning of the treatment. (A) Dermoscopy. (B) Illustration of the dermoscopic pattern “Focal structureless white areas with erythema pattern. The “Focal structureless white areas with erythema pattern” is composed of general erythema, focal structureless white areas, white lines, white dots, focal structureless yellow areas, polymorphic vessels (mostly glomerular vessels and doted vessels)

**Figure 6:**
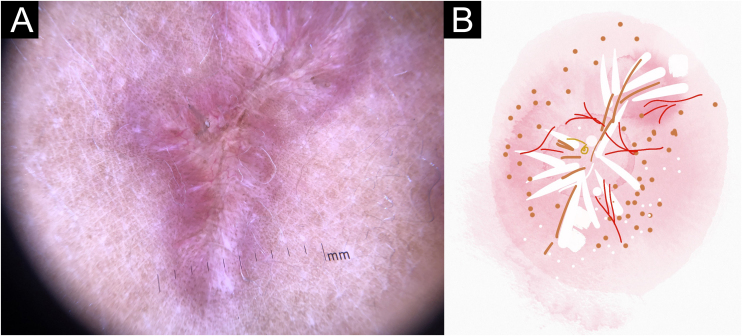
Sporotrichosis hypertrophic cicatricial lesion with central linear disposition of patient (n = 69) on the arm, at cure. (A) Dermoscopy. (B) Illustration of the dermoscopic pattern “white linear”. The “white linear pattern” is composed of general erythema, white lines, focal structureless white areas, white dots, black/brown dots, black lines, comedones and polymorphic vessels (mostly irregular linear vessels)

**Figure 7:**
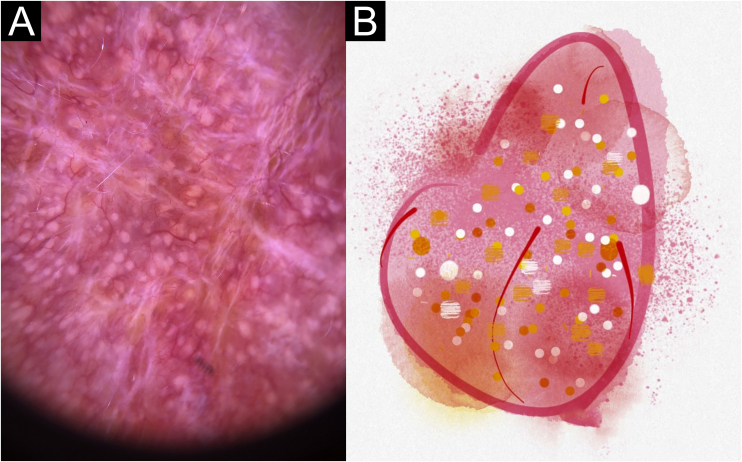
Sporotrichosis infiltrated plaque lesion on the face of patient (n = 21) at cure. (A) Dermoscopy. (B) Illustration of the dermoscopic pattern “strawberry”. The “strawberry pattern” is composed of general erythema with follicular plugs, white dots, and linear vessels

In this study, dermoscopic structures not yet related to CL and SP were observed (Supplementary Material): white lines, white dots, white perilesional circles, brown focal structureless areas, rosettes, rainbow patterns, brown dots, brown lines, inverted network, perilesional hyperchromic circle and comedon. Considering clinical and dermoscopic findings, the authors recognized the following global dermoscopic patterns (Table 3): at CL-T0 “central yellow scales with a white perilesional circle pattern” (Fig. 1), this pattern clinically corresponds to ulcerated or ulcer-verrucous lesions compatible with active disease, seen in 37 lesions (51%), especially in upper or lower extremities; in CL-T_1_ in 34 lesions (55%) “the diffuse structureless white area pattern” (white lines, focal structureless white areas, and white dots, brown focal structureless areas and hyperpigmented circle) (Fig. 2), this dermoscopic pattern corresponds to an epithelialized lesion, although there are still no signs of clinical cure; in CL-T_2_ “the white and brown focal structureless areas pattern” present in 44 lesions (32%) (white points and focal structureless white areas white lines, brown focal structureless areas and brown dots) (Fig. 3) pattern related to CL scar. At SP-T0, the authors found in 17 lesions (71%) the “pustule with erythema pattern” (Fig. 4). At SP-T_1_, the “focal structureless white areas with erythema pattern” was found in 10 lesions (63%) (erythema, focal structureless white areas, white dots, white lines, yellow areas, polymorphic vascular pattern, glomerular vessels, and linear vessels) (Fig. 5). At SP-T_2_, the “white linear” pattern was described in 10 lesions (56%) (focal structureless white areas, white dots, white lines, brown/black dots, brown focal structureless areas and brown lines) (Fig. 6). Patients with facial lesions (mostly plaque) had the “strawberry pattern” (erythema and follicular plugs with linear vessels) (Fig. 7); it was described in 5 lesions (7%) in CL-T_0_; in 4 lesions (7%) in CL-T_1_; in 2 lesions (3.3%) in CL-T_2_; and in 2 lesions (9%) in SP-T_0_.

**Table 3:** Dermoscopic patterns of Cutaneous Leishmaniasis Lesions (CL) and Sporotrichosis lesions (SP). Evandro Chagas National Institute of Infectious Diseases, Oswaldo Cruz Foundation, Rio de Janeiro, Brazil

Dermoscopic pattern	n (%)
CL-T_0_ Central yellow scales with white perilesional circle	37 (50.7)
CL-T_1_ Diffuse structureless white area pattern	34 (54.8)
CL-T_2_ White and brown focal structureless areas pattern	44 (31.7)
SP-T_0_ Pustule with erythema pattern	17 (70.8)
SP-T_1_ Focal structureless white areas with erythema pattern	10 (62.5)
SP-T_2_ White linear pattern	10 (55.6)

T_0_, Before the beginning of treatment; T_1_, Thirty days after the beginning of treatment; T_2_, After healing; CL, Cutaneous Leishmaniasis; SP, Sporotrichosis.

## Discussion

Unlike other dermoscopic studies of SP and CL, the authors studied the same lesions at different moments of the follow-up of the patients, which brought additional and new data.

Trying to avoid metaphoric terms, the authors based their descriptions on the standardization of dermoscopic terminology and basic dermoscopic parameters to evaluate in general dermatology (non-neoplastic dermatoses), made by the expert consensus on behalf of the International Dermoscopy Society (IDS),^9^ although there were some findings that the authors described as they appeared in the daily dermoscopic practice. Considering CL-T_0_, the authors observed two different dermoscopic patterns: 1) The “strawberry pattern” present in facial lesions, mostly plaques; and 2) The “central yellow scales with a white perilesional circle” predominantly observed in lesions in upper or lower extremities with ulceration and crust. This last pattern is very similar to the “white starburst” pattern previously described,^10^ but, many times the “starburst” was not present and was seen as a white perilesional circle. Regarding specific dermoscopic features at CL-T_0_, the authors found “yellow tears” only in 15%, unlike previous studies.^11^ The “salmon-colored ovoid structures” previously described^12^ were unusual in the present study (5% at CL-T_0_, 15% at CL-T_1_ and 21% at CL-T_2_), and were also the most difficult structure to differentiate since they can be confused with vascular structures, erythema, perilesional pigment and focal structureless areas. Their difficult differentiation and their low percentage of appearance at the occasion of diagnosis are enough reasons why they should not be considered as fundamental structures for the diagnosis of CL. The structure previously described as “yellow hue”^13^ was described in the present study as a focal structureless yellow area; it is indicative of the presence of granuloma in the dermis.^14^

The most common finding in the present study was erythema, present in 100% of SP-T_0_ and in 82% of CL-T_0_ (p = 0.034*). However, erythema is not a good healing criterion, because it was present in all evolutionary phases of both diseases. Erythema could be subdivided into dark, light and yellow^15^ but the authors could not detect relevant differences among the diseases and the points of follow-up, so they did not describe types of erythema. Regarding the vascular pattern, the present findings differed from a previous study,^10^ where comma vessels predominated (73%); the authors found they were relatively rare (2.8% at CL-T_0_, 13% at CL-T_1_, and 1.6% at CL-T_2_). However, the authors found polymorphic vessels pattern in 75% of CL-T_0_ cases, 39% of CL-T_1_ cases, and 35% of CL-T_2_ cases, similar to a previous study.^11^ The authors also observed dotted vessels in 56% at CL-T_0_, 37% at CL-T_1_, and 25% at CL-T_2_. According to other authors the dotted vessels were the predominant vascular structure in CL.^13^ The authors found glomerular vessels in 60% at CL-T_0_, 47% at CL-T_1_ and 28% at CL-T_2_. We must be careful in distinguishing between glomerular vessels and dotted vessels because they can be confused since glomerular vessels can be interpreted as punctiform vessels examined in a dermoscope (an increase of 10×), that’s why it has been suggested that most of the inﬂammatory diseases may display dotted vessels of variable diameter.^9^ The presence of pigmentary structures (brown focal structureless areas, dots and brown lines) may be also related to the high cutaneous phototype of the population since 49% of the patients had black-brown skin.

The dermoscopy of SP is much less described in the literature. A previous report^16^ was the case of a patient with disseminated SP, a rare clinical presentation, distinct from most of the patients with fixed ulcerated cutaneous SP with clinical suspicion of CL. Fixed cutaneous forms represent 20% to 30% of cases of SP^6^ and in this presentation, dermoscopy is a diagnostic tool to distinguish between SP and LC. The authors attributed distinct names to some dermoscopic structures previously described:^17^ they named the “white currents” as “focal structureless white areas” and the “hemorrhagic crusts” as “microulcerations”, these last ones are small contiguity solutions or exulcerations that cannot be seen with the naked eye but become apparent during the dermatoscopic examination. Dermoscopic findings described in SP-T_0_ as general erythema (100%), focal structureless white areas (87%), microulcerations (61%), central ulcer (65%), fiber sign (44%), pustules (39%) and focal structureless yellow areas (39%) are compatible with the clinical manifestations of SP, characterized by ulceration and exudation, that are associated with a more intense inflammatory process than observed in CL. Although white dots and white lines are observed in 48% of SP-T_0_, the presence of these structures increases progressively (68% and 70% respective) with the evolution towards a cure, which suggests that there is a direct relation between these findings and the process of fibrosis/healing. The presence of a white perilesional circle was observed mostly at SP-T_0_ (17%), suggesting it as a dermoscopy sign of an active lesion. At SP-T_1_, the authors observed a reduction in dermoscopic findings related to inflammation predominantly found in SP-T_0_, such as microulcerations, central ulceration, hyperkeratosis, and fiber sign; there was also an increase in structures related to fibrosis/healing (white points and lines), as well as the “pigmentary” structures (black-brown dots and brown focal structureless areas) predominantly visualized on SP-T_2_. At SP-T_2_, “pigmentary” dermoscopic findings increase, such as brown focal structureless areas (70%), black-brown dots (65%), inverted network (15%), and brown lines (15%). Concomitantly, at SP-T_2_, microulcerations, central ulcer, bleeding, pustules, fiber sign and white perilesional circle disappear. Finally, when comparing SP and CL the authors found that the most frequent dermoscopic findings (erythema, polymorphic vessel pattern, microulcerations, and focal structureless white areas) were common to both diseases and there were not pathognomonic or “speciﬁc clues” (features that are strongly suggestive of only one diagnosis, as they are related to highly speciﬁc histological ﬁndings).^9^ Although the sample size was not intended to show a statistical difference between the dermoscopic structures regarding both diseases, the authors observed a statistical difference in the presence of erythema, seen 100% in SP-T_0_ vs. 80% in CL-T_0_ (p = 0.034*) and in the presence of pustule, mostly seen in SP-T_0_ (p = 0.04*). So, erythema and pustule were findings that really helped guide the diagnosis and differentiate SP and CL on the first medical appointment. Even though the isolated analysis of dermoscopic structures is not enough to differentiate between the two diseases and none of the dermoscopic presentations were considered as speciﬁc clues; the authors showed previously undescribed dermoscopic patterns, which recognition might be very useful in the differential diagnosis between SP and CL. The overall pattern of SP was more asymmetric and less organized regarding the disposition of the dermoscopic structures when compared to CL, which in turn tended to present a radial distribution.

Considering the healing process, the architecture of SP scar differs greatly from the CL scar. There are two types of SP scars; the “candle drops like scar”,^18^ where the atrophic tissue is interspersed among hypertrophic tissue; and the linear scar, that are not related to the tension lines of the skin or to the original ulcer shape. Also, the comedon (40%) was an important and unique finding of SP scar differing from CL scar (0%). The comedon was a clinical and dermoscopic visible structure, that could express a cure for SP. In contrast, the typical CL scar usually has the same size and shape as the pre-existing ulcerative lesion, usually atrophic with a hypochromic central area and hyperchromic periphery.^19^ The presence of brown focal structureless areas, black/brown dots, brown lines, and inverted networks may be explained by the dyschromic process often observed in CL healing, and translates to the clinical aspects of a CL scar. Considering that brown dots, brown focal structureless areas, and the rainbow pattern, were all features predominated in T_2_, they could be interpreted as indicative of progression towards cure of the disease. The recognition of the clinical/dermoscopic aspects of CL scars is particularly important for epidemiological criteria purposes, especially if the authors consider the possibility of future mucosal lesions, one of the complications associated with ATL.^20^ The use of the manual dermoscope proved to be useful and practical, helping in the differential diagnosis between SP and CL, which is especially relevant in Rio de Janeiro state where both diseases coexist and have clinical similarities. In these circumstances, even small dermoscopic differences assume importance in the differential diagnosis between both diseases. Dermoscopy showed to be a tool in the differential diagnosis of these infectious diseases. Also, dermoscopy was useful in evaluating the clinical evolution and for monitoring the healing process towards the cure of these diseases.

In CL and SP, recent lesions usually present as ulcer, which tends, over time, to develop a vegetating or verrucous appearance. It is expected that different dermatological presentations have different dermoscopic aspects. One of the main limitations of this study is not being able to correlate dermoscopic presentations with the time of disease evolution at the first medical attendance.

## Conclusion

The authors provided a complete dermoscopic description of CL and SP in Latin America, describing different dermoscopic aspects of both diseases. The authors were also able to establish global dermoscopic patterns at different evolutionary moments of the diseases. Manual dermoscopy showed to be practical, useful, and easy to perform and contributed to the differential diagnosis, the clinical evolution, and for accompanying the healing process of CL and SP.

Special thanks to Dr. Dayvison Francis Saraiva Freitas for editing support. Dermatology Department. National Institute of Infectious Diseases- INI, Oswaldo Cruz Foundation, Rio de Janeiro, Brazil.

## Financial support

This study was partially supported by the Coordination for the Improvement of Higher Education Personnel (Coordenação de Aperfeiçoamento de Pessoal de Nível Superior ‒ CAPES). It was also funded by National Council for scientific and technological development (Conselho Nacional de Desenvolvimento Científico e Tecnológico: CNPq; [grant number CMVR 313327/2018-1]). The funders had no role in study design, data collection and analysis, the decision to publish, or the preparation of the manuscript.

## Authors' contributions

Alejandra Galeano España: Contributed significantly to the conception and design of the study; data collection; analysis and interpretation of data; statistical analysis; article writing or critical review of important intellectual content; collection, analysis, and interpretation of data; effective participation in research guidance; intellectual participation in propaedeutic and/or therapeutic conduct of case studies; critical review of the literature; final approval of the final version of the manuscript.

Maria Inês Fernandes Pimentel: Contributed significantly to the conception and design of the study; data collection; analysis and interpretation of data; statistical analysis; article writing or critical review of important intellectual content; collection, analysis and interpretation of data; effective participation in research guidance; intellectual participation in propaedeutic and/or therapeutic conduct of case studies; critical review of the literature; final approval of the final version of the manuscript.

Janine Pontes de Miranda Lyra: Contributed significantly to the conception and design of the study; analysis and interpretation of data; article writing or critical review of important intellectual content; collection, analysis, and interpretation of data; critical review of the literature; final approval of the final version of the manuscript.

Cláudia Maria Valete-Rosalino: Contributed significantly to the conception and design of the study; data collection; analysis and interpretation of data; statistical analysis; collection, analysis and interpretation of data; effective participation in research guidance; Intellectual participation in propaedeutic and/or therapeutic conduct of case studies; final approval of the final version of the manuscript.

Marcelo Rosandiski Lyra: Contributed significantly to the conception and design of the study; data collection; analysis and interpretation of data; statistical analysis; article writing or critical review of important intellectual content; collection, analysis and interpretation of data; effective participation in research guidance; intellectual participation in propaedeutic and/or therapeutic conduct of case studies; critical review of the literature; final approval of the final version of the manuscript.

## Conflicts of interest

None declared.

## Figures and Tables

**Figure:**
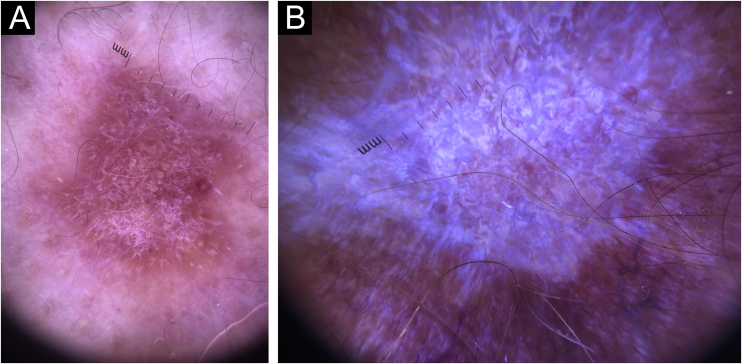


**Figure:**
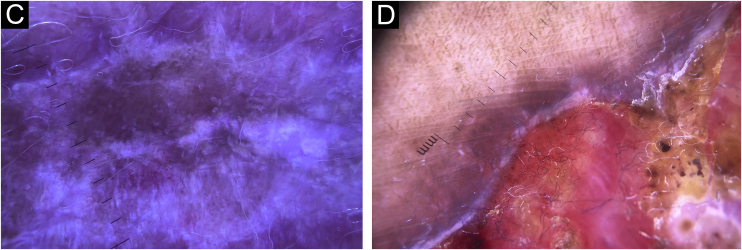


**Figure:**
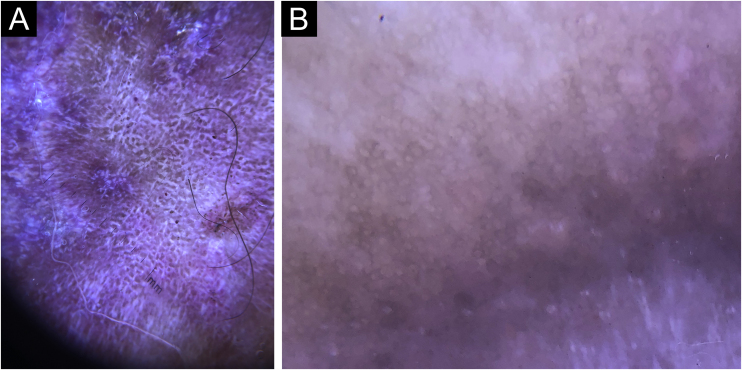


**Figure:**
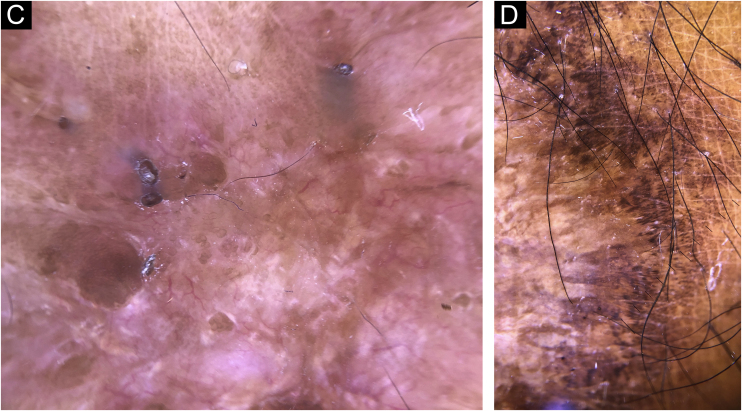


## Footnotes

* means statistical difference.
