# Supplementary material for: Description of the dermatoscopic features observed in sporotrichosis and American cutaneous leishmaniasis in a reference center in Rio de Janeiro, Brazil
Source: An Bras Dermatol. 2023 Jul 20;98(6):764–73. doi: 10.1016/j.abd.2022.09.015 (PMC10589486; doi:10.1016/j.abd.2022.09.015)
Supplement: Supplementary file 1 [file mmc1.docx]

ABD-D-22-00306_Supplementary material

Other dermatoscopic features

**Figure Supplementary 1** CL and SP new dermoscopic features related. (A)White lines (arrow), white dots (circule); (B) Rainbow pattern.

**Figure Supplementary 2** CL and SP new dermoscopic features related. (A) Rosettes (white keratotic plugs); (B) white perilesional circle.

**Figure Supplementary 3** CL and SP new dermoscopic features related. (A) Inverted network. (B) Brown focal structureless areas.

**Figure Supplementary 4** CL and SP new dermoscopic features related. (C) Comedones (D) Perilesional hyperchromic circle, form by brown dots (circule) & lines (arrow).
